# Evaluation of a point-of-care diagnostic to identify glucose-6-phosphate dehydrogenase deficiency in Brazil

**DOI:** 10.1371/journal.pntd.0009649

**Published:** 2021-08-12

**Authors:** Stephanie Zobrist, Marcelo Brito, Eduardo Garbin, Wuelton M. Monteiro, Suellen Clementino Freitas, Marcela Macedo, Aline Soares Moura, Nicole Advani, Maria Kahn, Sampa Pal, Emily Gerth-Guyette, Pooja Bansil, Gonzalo J. Domingo, Dhelio Pereira, Marcus VG Lacerda

**Affiliations:** 1 Diagnostics, PATH, Seattle, Washington, United States of America; 2 Fundação de Medicina Tropical Dr Heitor Vieira Dourado (FMT/HVD), Manaus, Amazonas, Brazil; 3 Universidade do Estado do Amazonas, Manaus, Amazonas, Brazil; 4 Centro de Pesquisa Em Medicina Tropical (CEPEM), Porto Velho, Rondônia, Brazil; 5 Universidade Federal de Rondônia (UNIR), Porto Velho, Rondônia, Brazil; 6 Instituto Leônidas & Maria Deane (ILMD), Fiocruz, Manaus, Amazonas, Brazil; Academic Medical Center: Amsterdam UMC Locatie AMC, NETHERLANDS

## Abstract

**Background:**

Glucose-6-phosphate dehydrogenase (G6PD) deficiency is a common enzyme deficiency, prevalent in many malaria-endemic countries. G6PD-deficient individuals are susceptible to hemolysis during oxidative stress, which can occur from exposure to certain medications, including 8-aminoquinolines used to treat *Plasmodium vivax* malaria. Accordingly, access to point-of-care (POC) G6PD testing in Brazil is critical for safe treatment of *P*. *vivax* malaria.

**Methodology/Principal findings:**

This study evaluated the performance of the semi-quantitative, POC STANDARD G6PD Test (SD Biosensor, Republic of Korea). Participants were recruited at clinics and through an enriched sample in Manaus and Porto Velho, Brazil. G6PD and hemoglobin measurements were obtained from capillary samples at the POC using the STANDARD and HemoCue 201+ (HemoCue AB, Sweden) tests. A thick blood slide was prepared for malaria microscopy. At the laboratories, the STANDARD and HemoCue tests were repeated on venous samples and a quantitative spectrophotometric G6PD reference assay was performed (Pointe Scientific, Canton, MI). G6PD was also assessed by fluorescent spot test. In Manaus, a complete blood count was performed.

Samples were analyzed from 1,736 participants. In comparison to spectrophotometry, the STANDARD G6PD Test performed equivalently in determining G6PD status in venous and capillary specimens under varied operating temperatures. Using the manufacturer-recommended reference value thresholds, the test’s sensitivity at the <30% threshold on both specimen types was 100% (95% confidence interval [CI] venous 93.6%–100.0%; capillary 93.8%–100.0%). Specificity was 98.6% on venous specimens (95% CI 97.9%–99.1%) and 97.8% on capillary (95% CI 97.0%–98.5%). At the 70% threshold, the test’s sensitivity was 96.9% on venous specimens (95% CI 83.8%–99.9%) and 94.3% on capillary (95% CI 80.8%–99.3%). Specificity was 96.5% (95% CI 95.0%–97.6%) and 92.3% (95% CI 90.3%–94.0%) on venous and capillary specimens, respectively.

**Conclusion/Significance:**

The STANDARD G6PD Test is a promising tool to aid in POC detection of G6PD deficiency in Brazil.

**Trial registration:**

This study was registered with ClinicalTrials.gov (identifier: NCT04033640).

## Author summary

G6PD deficiency affects an estimated 500 million people worldwide and is prevalent in many malaria-endemic settings. People with G6PD deficiency are at risk of hemolysis when exposed to certain medications, including 8-aminoquinoline drugs used to treat *Plasmodium vivax* malaria. Increased access to testing for G6PD deficiency at or near the point of care is critical for expanding the safe treatment of *P*. *vivax* malaria. In this study, we aimed to evaluate the performance of a point-of-care, semi-quantitative test for G6PD deficiency, the STANDARD G6PD Test, in a malaria-endemic setting in Brazil. The test was evaluated on both capillary and venous blood samples across a broad range of operating temperatures. The findings show that the STANDARD G6PD Test performed equivalently to the reference test in its ability to diagnose G6PD deficiency at the point of care. The STANDARD G6PD Test is a promising tool to aid in detecting G6PD deficiency at the point of care in Brazil.

## Introduction

Glucose-6-phosphate dehydrogenase (G6PD) deficiency is a common genetic condition, with over 180 observed variants globally [1]. It affects approximately 500 million individuals worldwide and is prevalent in malaria-endemic settings [2,3,4]. In Brazil, where malaria remains a serious public health concern, the prevalence of G6PD deficiency is approximately 5%, but reported estimates range up to 12.9% among some populations [3,5].

The G6PD enzyme produces the reduced form of nicotinamide adenine dinucleotide phosphate that supports red blood cells in protecting against oxidative challenge [2]. When G6PD activity is reduced, these cells are more vulnerable to oxidant hemolysis upon exposure to certain exogenous agents, including some foods, diseases, and drugs [6]. Because G6PD is an X-linked genetic disorder, males can be either hemizygous G6PD deficient or normal by phenotype. Females, however, can be either homozygous or heterozygous for the G6PD gene and therefore exhibit a broader range of deficiencies due to random inactivation of one of the two X chromosomes (lyonization) [1,7]. According to current World Health Organization (WHO) case definitions, males and females with G6PD activity levels of <30% of normal as determined by the population median are considered to be G6PD deficient, heterozygous females with activity levels between 30% and 80% are considered to be G6PD intermediate, and males with >30% activity levels and females with >80% activity levels are considered to be G6PD normal [8].

Primaquine and tafenoquine (8-aminoquinoline drugs) are used to eliminate dormant liver-stage hypnozoites and prevent recurrent infection from *Plasmodium vivax (P*. *vivax)* malaria. Patients with G6PD deficiency are at increased risk of acute hemolytic events following exposure to these drugs, which limits their broader clinical use [9]. As such, the availability of point-of-care testing for G6PD deficiency greatly benefits increased safe access to 8-aminoquinolines [10]. The current WHO malaria treatment guidelines recommend that G6PD testing be conducted prior to prescription of primaquine [11]. However, in practice, the G6PD status of patients is often unknown at the time of malaria diagnosis due to the price and complexity of the available diagnostic products—especially in the context of malaria-endemic and low-resource settings [12,13]. In such cases, WHO advises that an assessment of the potential risks and benefits of primaquine administration should be used to inform clinical decision-making [11]. Further, tafenoquine (Krintafel/Kozenis; marketed under the brand name Kozenis in Brazil), which is administered in a single-dose format, is indicated only for patients with >70% G6PD activity, necessitating the use of a quantitative or semi-quantitative test prior to prescription [14,15]. As such, appropriate methods for G6PD diagnostic testing at or near where patients seek care for malaria are essential to guide safe and effective management of *P*. *vivax* cases and increase the potential of these medications to support malaria control and elimination efforts [10].

The reference standard for measurement of G6PD activity is a quantitative UV spectrophotometric assay [16,17]. This test is complex, requiring laboratory infrastructure (e.g., equipment, electricity, fluorescent light) and experienced personnel to implement—making it inaccessible for most near-patient use cases in malaria-endemic settings. The most widely used test is the qualitative fluorescent spot test, which is less expensive and simpler to operate. However, there are still notable limitations with this test, which requires a cold chain, some laboratory infrastructure, and trained personnel. To fill this diagnostic gap and bring testing for G6PD deficiency closer to the patient, novel point-of-care tests are becoming increasingly available [18,19]. These tests include quantitative or semi-quantitative biosensors that provide a numerical measurement of enzyme activity on a digital reader [20,21,22], as well as qualitative rapid diagnostic tests (RDTs) that provide binary or categorical assessments of G6PD status that are interpreted visually [19]. Importantly, qualitative RDTs are only able to identify severe deficiencies (<30% activity), whereas quantitative or semi-quantitative biosensors can discriminate between the full range of deficiencies and therefore identify heterozygous women with intermediate G6PD status [19].

To date, Brazil’s public health system has not implemented routine screening to identify individuals with G6PD deficiency [23]. However, an increasing proportion of *P*. *vivax* cases among all malaria cases in the country underscores the importance of testing for G6PD deficiency at the point of care in Brazil [24,25]. In a recent economic analysis, Peixoto et al. (2016) estimate that the use of primaquine among the population with G6PD deficiency represents a significant burden for Brazil’s public health system [26]. Brazil’s recently revised malaria treatment guidelines (2020) recommend that patients with *P*. *vivax* malaria be tested for G6PD status prior to primaquine administration, if testing is available. Additionally, quantitative testing is also advised prior to prescription of Kozenis, which is being gradually implemented in Brazil [27].

SD Biosensor (Republic of Korea) has developed a novel, semi-quantitative point-of-care G6PD test: the STANDARD G6PD Test. This test is an enzymatic colorimetric assay intended to aid in the detection of G6PD deficiency. It measures G6PD enzymatic activity and total-hemoglobin (T-Hb) concentration in fresh capillary and venous human whole blood specimens. The test components include a portable, battery-operated analyzer, disposable test strips called Test Devices, individual extraction buffer tubes, sample collector tubes called EziTubes+, and a lot-specific code chip that is used to calibrate the analyzer. To conduct the test, a blood sample is collected using an EziTube+, mixed with the extraction buffer solution, and applied to the Test Device, which is then inserted into the analyzer. After two minutes, the analyzer provides a numeric measurement of G6PD activity normalized by hemoglobin (U/g dL), as well as T-Hb (g/dL). The G6PD measurement output can then be used in a semi-quantitative manner to classify individuals as G6PD deficient, intermediate, or normal according to thresholds provided by the manufacturer. A Quick Reference Guide that summarizes the test procedures is included as S1 Fig and is also available at https://www.path.org/programs/diagnostics/gorcop/.

The primary objective of this study was to evaluate the performance of the STANDARD G6PD Test as compared to a spectrophotometric reference assay in the detection of G6PD activity when used by trained health care workers in a malaria-endemic setting in Brazil. A secondary objective was to evaluate the performance of the test in the measurement of hemoglobin concentration.

## Methods

### Ethics statement

Ethical approval for this study was obtained from the PATH Research Ethics Committee, the Ethics Committee Board of *FMT/HVD* (approval number 94833618.0.1001.0005), Brazil’s National Research Ethics Commission (CONEP; approval number 94833618.0.1001.0005), and the CEPEM ethics committee (approval number 94833618.0.2001.0011). Written informed consent to participate in the study was obtained for all participants. For participants under 18 years of age, parents provided written consent for their child to participate. In addition to parental consent, children between the ages of 5 and 17 also provided written assent to participate.

### Study site and population

This study was conducted at two sites in Brazil: the *Fundação de Medicina Tropical Dr Heitor Vieira Dourado* (FMT/HVD) in Manaus, Amazonas, and the *Centro de Pesquisa em Medicina Tropical de Rondônia* (CEPEM) in Porto Velho, Rondônia. Manaus has an estimated population of more than 2 million people, compared to approximately 520,000 people in Porto Velho [28]. Both sites are situated in the Brazilian Amazon, which accounts for the vast majority of the country’s malaria cases [23,29,30]. Both FMT/HVD and CEPEM serve as reference centers for malaria diagnosis and treatment in their respective states.

Participants included febrile patients seeking care at the Manaus and Porto Velho clinics. To ensure sufficient representation of G6PD activity levels across the dynamic range, an enriched sample of individuals from the surrounding communities with known G6PD status, established through previous epidemiological studies, was also included. At each site, a list of 180 individuals with known G6PD status from prior epidemiological studies who agreed to be contacted for future research was generated. Using this list, the study employed a block randomization scheme to generate 30 blocks of six individuals each, with a 1:1:1 ratio of G6PD normal, deficient, and intermediate status. Study staff were provided the randomization scheme as a recruitment list, and participants from the enriched sample were recruited through home visits. This method was employed to ensure that study personnel performing point-of-care testing were blinded to the G6PD status of all enrolled participants. Individuals two years of age and older were eligible to participate in the study. Individuals who self-reported receiving a blood transfusion in the past three months were not eligible.

### Study design

A cross-sectional diagnostic accuracy study was conducted in Manaus and Porto Velho, Brazil, between July and December of 2019. Based on the expected prevalence of G6PD deficiency at the sites (5%) [3,5], the study aimed to recruit a minimum of 100 G6PD-deficient cases.

The performance of the STANDARD G6PD Test on both capillary and venous specimens was compared to matching G6PD values from venous specimens tested with a spectrophotometric reference assay normalized by hemoglobin. The performance of the STANDARD G6PD Test in the measurement of hemoglobin concentration was compared to a complete blood count from an automated hematology analyzer (Sysmex KX-21N) in Manaus, as well as to results from venous specimens on the HemoCue 201+ system (HemoCue AB, Ängelholm, Sweden) in both Manaus and Porto Velho. Table 1 summarizes the tests included in the study.

**Table 1:** Summary of study tests.

Location	Clinical site and reference testing facility	Point-of care tests (capillary specimens)			Laboratory tests (venous specimens)				
		G6PD activity (U/g Hb)	Malaria	Hemoglobin (g/dL)	Reference G6PD activity (U/g Hb)	Reference hemoglobin (g/dL)	G6PD activity (U/g Hb)	Comparison G6PD	Comparison hemoglobin (g/dL)
Manaus, Amazonas, Brazil	FMT/HVD	STANDARD G6PD Test	Microscopy (thick blood slide)	HemoCue Hb 201+	Pointe Scientific G6PD reagent kit (Cat No. G7583) on a Shimadzu six-cell, temperature-regulated manual spectrophotometer (UV-1800)	CBC (Sysmex KX-21N)*	STANDARD G6PD Test	FST (Trinity Biotech G-6-PDH Deficiency Screen by Spot Test)	HemoCue Hb 201+
Porto Velho, Rondônia, Brazil	CEPEM					HemoCue Hb 201+*			-

*Used for G6PD normalization

CBC, complete blood count; CEPEM, Centro de Pesquisa em Medicina Tropical de Rondônia; FMT/HVD, The Fundação de Medicina Tropical Dr Heitor Vieira Dourado; FST, fluorescent spot test; G6PD, glucose-6-phosphate dehydrogenase; UV, ultraviolet

### Study procedures at the point of care

After obtaining informed consent, demographic information was collected from participants using standard questionnaires. G6PD and hemoglobin measurements were obtained directly from non-anticoagulated capillary fingerstick blood samples using the STANDARD G6PD and HemoCue tests. A thermometer and a hygrometer were placed near the point-of-care tests at the time of testing to collect temperature and humidity data. Point-of-care tests were performed prior to the reference tests and by staff blinded to the G6PD status of participants. Additionally, a thick blood slide was prepared using capillary blood for malaria microscopy. Slides were read by trained microscopists and used to inform malaria diagnosis, as per the standard of care at both study sites. Four mL of venous blood was obtained from participants using a standard venipuncture kit and collected in a K_2_EDTA-treated tube. Immediately after collection, specimens were stored in a refrigerator at the clinic or cooler box at 4 to 6°C and transferred to the laboratory. Refrigerator temperature monitoring was conducted daily.

### Laboratory testing procedures

At the laboratories, aliquots of venous blood were used to run further G6PD and hemoglobin tests as described below. Microscopic examinations of thick blood slides were conducted to determine malaria diagnosis. Thermometers and hygrometers were also placed near the assays in the laboratory at the time of data collection. All laboratory test operators were blinded to the results of the point-of-care tests, and operators of the reference tests were blinded to the results of the replicate STANDARD G6PD Test that had been run on venous specimens in the laboratory.

#### Spectrophotometric G6PD reference assay

The quantitative G6PD reference assay was run in duplicate using the Pointe Scientific reagent kit (Pointe Scientific, Canton, MI; Cat No. G7583) on a Shimadzu UV-1800 six-cell temperature-regulated manual spectrophotometer set to 37°C in accordance with manufacturer instructions. Testing was done within 72-hours of specimen collection. Each day, prior to clinical testing, commercially available G6PDH deficient, intermediate, and normal controls were run on the machines (Analytical Control Systems, Fishers, IN; Cat. Nos. HC-108, HC-108IN, and HC-108DE).

#### Fluorescent spot test (FST)

Each specimen was also tested using the Trinity qualitative G6PD FST kit (Trinity Biotech, Bray, Ireland, G-6-PDH Screen by Spot Test; Cat No. 203-A) to represent a widely used, standard-of-care test for G6PD deficiency. The test was conducted according to manufacturer instructions. It was used each day in conjunction with commercially available controls (Trinity Biotech; Cat Nos. G5888, G5029, G6888).

#### STANDARD G6PD test

G6PD activity normalized by hemoglobin (Hb) was also measured with the STANDARD G6PD Test on venous specimens in the laboratory, as per the manufacturer’s instructions. For testing with venous specimens, 10 μL of venous blood was transferred into the test’s buffer tube using a professional pipette. The specimen was then mixed with the buffer 10 to 15 times using the pipette, and 10 μL of the mixed specimen was transferred onto the device’s test strip using a new pipette tip. Results were obtained within two minutes. The test reports results in U/g Hb for G6PD activity and g/dL for hemoglobin concentration. The devices used in this study were indicated to function at an operating temperature of 15°C to 40°C and across a dynamic range of 0 to 20 U/g Hb for G6PD and 4 to 25 g/dL for hemoglobin. As with the tests used at the point of care, the manufacturer’s quality controls were run on the devices each day prior to clinical testing.

The manufacturer of the STANDARD G6PD Test (SD Biosensor) has established single thresholds for G6PD activity (U/g Hb) for the test’s classification of G6PD status on both capillary and venous specimens based on previous published and unpublished clinical data [21,22]. The thresholds recommended by the manufacturer are: ≤4.0 U/g Hb for G6PD-deficient males and females (≤30% activity), 4.1 to 6.0 U/g Hb for G6PD-intermediate females (>30% to ≤70% activity), >4.0 U/g Hb for G6PD-normal males (>30% activity), and >6.0 U/g Hb for G6PD-normal females (>70% activity).

#### Complete blood count (CBC)

In Manaus, hemoglobin concentration was determined by CBC within 24 hours using an automated hematology analyzer (Sysmex KX-21N). Results of the CBC were used to normalize the spectrophotometric reference test results in Manaus.

#### HemoCue 201+

At both sites, the HemoCue 201+ test was run on aliquoted venous blood in the laboratory within 24 hours of sample collection. In Porto Velho, this result was used to normalize the site’s spectrophotometric reference test results.

### Follow-up

Participants who tested positive for malaria or anemia at the point of care were counseled and referred for follow-up according to the standard of care in Brazil. All participants who tested G6PD intermediate or deficient by the reference assay received follow-up counseling with their test results. Participants found to be *P*. *vivax* positive and who tested G6PD deficient or intermediate with the STANDARD G6PD Test at the point of care were referred to the health system for care with the recommendation that primaquine be withheld or closely monitored based on their level of G6PD activity until confirmatory testing could be completed.

### Data management

All study results were recorded on data collection forms. Data was entered into REDCap (Research Electronic Data Capture) [31], a secure, web-based software platform, and was routinely monitored for accuracy in relation to source documents.

### Statistical methods

The performance of the STANDARD G6PD Test against the spectrophotometric reference test was determined by calculating the test’s sensitivity and specificity as described in Domingo et al. (2013) [32]. Absolute G6PD values on the reference assay were normalized at each site to facilitate inter-laboratory comparison [33]. Reference values were expressed as the percentage of each site’s adjusted male median determined from 36 randomly selected males with G6PD activity within the G6PD reference range for the reference assay in all males established at each laboratory. These were subsequently excluded from the performance analysis [14,34]. Quantitative thresholds for G6PD activity categories at 30%, 70%, and 80% were then calculated and used to assess performance between the STANDARD G6PD Test and the reference test. Sensitivity and specificity were calculated using 95% confidence intervals.

Receiver operating characteristic (ROC) curves were plotted at 30%, 70%, and 80% activity thresholds. Quantitative agreement for G6PD activity between the STANDARD G6PD Test and the reference test was analyzed by linear regression. Correlation graphs between the STANDARD G6PD Test results and the reference test were plotted, and R-squared values were determined. Bland-Altman plots were also generated to analyze bias. In addition, the agreement between the reference test and the STANDARD G6PD Test was determined. Kappa coefficients were also calculated to assess agreement. Similar analyses were performed between the STANDARD G6PD Test’s hemoglobin values and reference hemoglobin values.

For the qualitative analysis of the STANDARD G6PD Test performance, results are presented according to the clinically relevant thresholds used to inform the administration of 8-aminoquinolines: G6PD-deficient cases were defined as males and females with 30% or less G6PD activity, and females with intermediate G6PD activity were defined as those with greater than 30% activity and less than or equal to 70% activity. Additional analyses at the 80% intermediate threshold are presented in supplemental materials.

All statistical analyses were performed using Stata 15.0 (StataCorp, College Station, TX).

## Results

### Study population

In total, 1,736 individuals were included in the study: 924 from Manaus and 812 from Porto Velho (Table 2). Of these, 69 participants were enrolled through the enriched sample: 60 in Manaus and 9 in Porto Velho. Venous samples were available from 1,662 participants, and capillary samples were available from 1,693 participants. If controls failed on a test prior to its use in testing clinical samples, associated data were excluded from the analysis.

**Table 2:** Characteristics of the study population.

	Manaus	Porto Velho	Combined
**Final analytic population, n**	924	812	1,736
**Recruited through enriched sample, n**	60	9	69
**Age (years)**			
Mean (SD)	36.1 (12.8)	39.6 (15.0)	37.7 (13.9)
Range	5–84	2–92	2–92
**Age categories, n (%)**			
2–11 years	6 (0.7)	18 (2.2)	24 (1.4)
12–15 years	2 (0.2)	20 (2.5)	22 (1.3)
16–64 years	896 (96.1)	736 (90.6)	1,632 (94.0)
65+ years	20 (2.2)	38 (4.7)	58 (3.3)
**Sex, n (%)**			
Female	530 (57.4)	418 (51.5)	948 (54.6)
Male	394 (42.6)	394 (48.5)	788 (45.4)
**G6PD status** * **, n (%)**			
Deficient	30 (3.3)	29 (3.6)	59 (3.4)
Intermediate	14 (1.5)	21 (2.6)	35 (2.0)
Normal	880 (95.2)	762 (93.8)	1,642 (94.6)
**Anemia status** ^**‡**^ **, n (%)**			
Non/mild	853 (92.3)	758 (93.4)	1,611 (92.8)
Moderate	60 (6.5)	51 (6.3)	111 (6.4)
Severe	11 (1.2)	3 (0.4)	14 (0.8)
**Febrile** ** **, n (%)**			
Female	6 (0.7)	30 (3.7)	36 (2.1)
Male	11 (1.2)	50 (6.2)	61 (3.5)
**Malaria confirmed cases** ^**§**^ **, n (%)**	14 (1.5)	237 (29.2)	251 (14.5)
**Malaria status**			
*P*. *falciparum* positive	2 (0.22)	10 (1.2)	12 (0.7)
*P*. *vivax* positive	12 (1.30)	187 (23.0)	199 (11.5)
Both *P*. *falciparum* and *P*. *vivax* positive	0 (0)	40 (4.9)	40 (2.3)
Negative	909 (98.4)	575 (70.8)	1,484 (85.5)

SD, standard deviation; *P falciparum*, *Plasmodium falciparum*; *P*. *vivax*, *Plasmodium vivax*; G6PD, glucose-6-phosphate dehydrogenase.

* G6PD status as determined by the spectrophotometric reference G6PD test, based on 30% and 70% thresholds.

** Defined as having a temperature of ≥37.5°C at enrollment [40].

‡ Anemia status in accordance with published clinically relevant hemoglobin concentration thresholds from the World Health Organization (WHO), as measured by HemoCue on venous specimens [35]. Non/mild anemia categories have been combined due to the clinical significance of a moderate or severe anemia diagnosis.

§ Microscopy positive at recruitment.

Overall, the mean age of participants was 37.7 years, ranging from 2 to 92 years. The majority of participants were between 16 and 64 years of age (94.0%), were female (54.6%), had no or mild anemia (92.8%), and were malaria negative (85.5%). Although the distribution of most of these characteristics was similar across both sites, the majority (94.4%) of the malaria-positive cases were found in Porto Velho.

### G6PD activity distribution in the study population

The adjusted male median, or 100% G6PD activity, by the spectrophotometric reference assay from the randomly selected 36 males at each site was 8.6 U/g Hb for Manaus (95% CI 8.1–9.2) and 8.9 U/g Hb for Porto Velho (95% CI 8.6–9.4). There was no significant difference observed between the adjusted male medians between sites (P = 0.25). There was also no significant difference between the adjusted male median of these 36 males and that of the whole study normal male population. The adjusted male median for all normal males in Manaus was 8.9 U/g Hb (95% CI 8.3–9.5), with a *P* value of 0.49, and in Porto Velho the same two values were 9.1 U/g Hb (95% CI 8.3–10.1) and 0.18, respectively. In Porto Velho, where there was a sufficient number of malaria cases among normal males to assess the variance from the whole male normal population, a slight decrease was observed in the adjusted male median among malaria positive normal males (8.7 U/g Hb 95% CI 8.0–9.7) compared to all normal males, with a *P* value of 0.004 but with significantly overlapping confidence intervals. Using the adjusted male median activities, the 30% activity threshold corresponded to 2.58 U/g Hb and 2.67 U/g Hb for Manaus and Porto Velho, respectively. The 70% activity threshold corresponded to 6.02 U/g Hb for Manaus and 6.23 U/g Hb for Porto Velho. Based on these thresholds, there were a total of 59 G6PD-deficient participants and 35 G6PD-intermediate participants in the study sample (Table 2).

**Fig 1:**
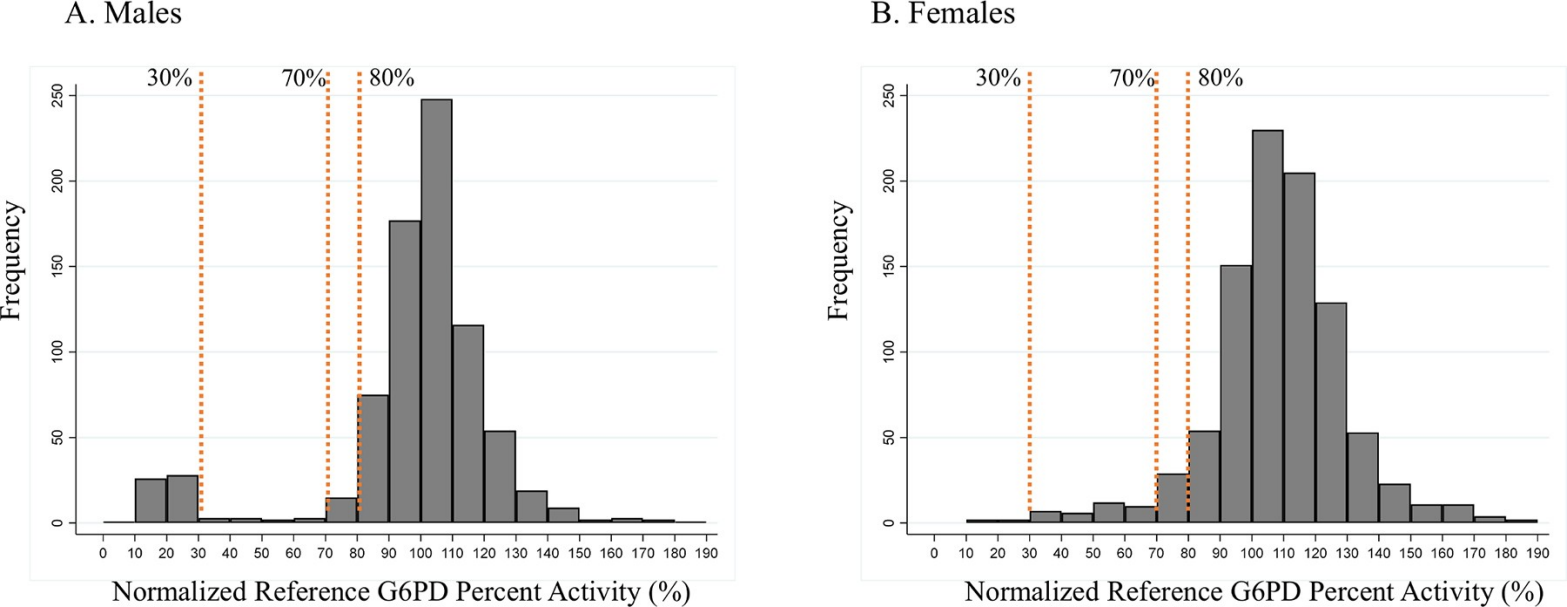
Histogram showing the distribution of G6PD activities in the study population. **The frequency is plotted against the normalized reference assay G6PD activity in 10% increments for A) males, and B) females. The 30%, 70%, and 80% activity limits are indicated on each plot.** G6PD, glucose-6-phosphate dehydrogenase.

The adjusted male median was used to normalize the reference G6PD activities at each site on a percent activity scale. Fig 1 shows the distribution of G6PD activity of the study population in percent activity, by sex. These figures show a clear bimodal distribution for male participants as compared to a continuous distribution for females, consistent with the epidemiology of G6PD activity. The number of females and males within intermediate activity ranges between 50% and 80%, as measured by the spectrophotometric reference test and the FST, are presented in Table 3. Of note, in the 70% to 80% activity range, there are 15 males that are most likely hemizygous G6PD normal. In contrast, there are 29 females in this activity range as measured by the reference assay. Of these 44 participants, 95% (42/44) were classified as G6PD normal by FST. In the 60% to 70% activity range, there were 10 females and 3 males. Of these 13 individuals, nine FST test results were normal (69%). Between 60% and 70%, the ratio of females to males was greater, and the ratio of females classified as intermediate by FST was also greater.

**Table 3:** Number of females and males within intermediate G6PD activity ranges (50 to 80%). The fluorescent spot test (FST) results that are available are also shown. The results are provided in terms of normal, intermediate, and deficient (N:I:D).

Percent activity range as determined by the spectrophotometric reference assay	Female		Male	
	Reference assay result, n	FST result, n N:I:D	Reference assay result, n	FST result, n N:I:D
≥70% and <80%	29	29 27:2:0	15	15 15:0:0
≥60% and <70%	10	10 7:3:0	3	3 2:1:0
≥50% and <60%	12	12 1:10:1	2	2 1:1:0

G6PD, glucose-6-phosphate dehydrogenase; FST, fluorescent spot test; N:I:D, normal: intermediate: deficient.

### Operating temperature for the STANDARD G6PD Test both at the point of care and in the laboratory

Enzymatic assays are sensitive to variations in temperature and humidity [32]. It is important that point-of-care tests used to support malaria case management are evaluated under real-life environmental conditions. During the study, the STANDARD G6PD Test was run over a broad range of operating temperatures, ranging from 17.7°C to 43.7°C (S1 Table), which is representative of true operating conditions for the device in a real-world, malaria-endemic setting.

### Clinical performance of the STANDARD G6PD Test for G6PD activity

**Fig 2:**
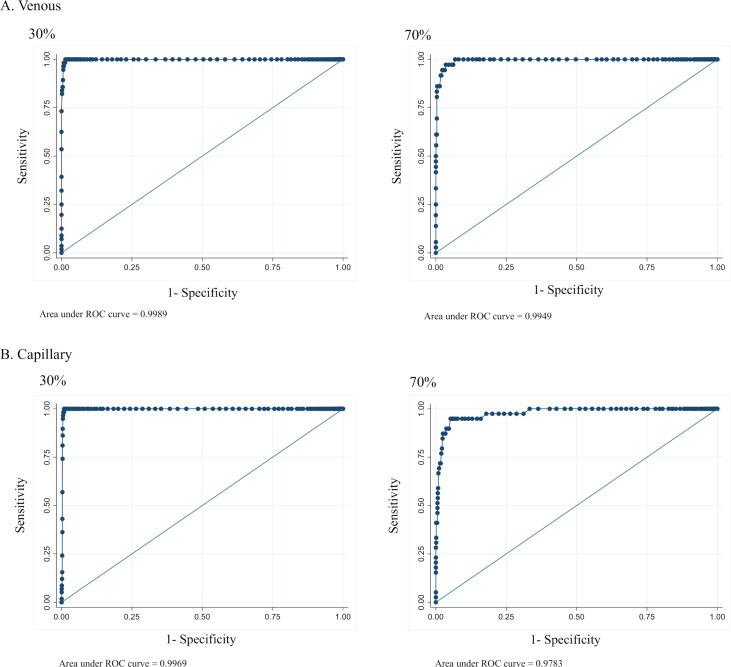
**ROC curves at 30% and 70% G6PD activity thresholds on A) venous specimens and B) capillary specimens.** ROC, receiver operating curve.

ROC curves were generated to assess the ability of the STANDARD Test to discriminate G6PD-deficient males and females (0% to 30% G6PD activity), G6PD-intermediate females (30% to 70% G6PD activity), and G6PD-normal males (>30% activity) and females (>70% activity) (Fig 2). ROC curves for the 80% intermediate activity threshold are presented in supplemental materials (S2 Fig). Associated performance characteristics are provided in S2 Table, by specimen type. The test was able to diagnose G6PD-deficient males and females with a high area under the curve (AUC) of 1.0 across both capillary and venous specimens. At the 70% threshold for intermediate females, the test also showed a high AUC at 1.0 and 0.98 for venous and capillary specimens, respectively. At the 80% threshold for intermediate females, the test showed a drop in AUC for both specimen types: 0.95 for venous and 0.90 for capillary. Overall, the STANDARD G6PD Test was able to provide robust classifications of G6PD activity across the dynamic range, with a drop in AUC at 80%.

Sensitivity and specificity of the STANDARD G6PD Test were calculated based on the manufacturer’s thresholds for G6PD deficient, intermediate, and normal (Tables 4 and S3). Corresponding 2x2 contingency tables are presented in the supplemental materials (S4 Table). For G6PD-deficient males and females, the sensitivity of the test on both venous and capillary specimens was 100% (95% CI venous 93.6%–100.0%; capillary 93.8%–100.0%), whereas specificity was 98.6% on venous specimens (95% CI 97.9%–99.1%) and 97.8% on capillary specimens (95% CI 97.0%–98.5%). For intermediate females, the test’s sensitivity was 96.9% on venous specimens (95% CI 83.8%–99.9%) and 94.3% on capillary (95% CI 80.8%–99.3%). Specificity was 96.5% (95% CI 95.0%–97.6%) and 92.3% (95% CI 90.3%–94.0%) on venous and capillary specimens, respectively.

**Table 4:** Diagnostic performance of the STANDARD G6PD Test using the manufacturer’s thresholds compared with normalized spectrophotometric reference values, by specimen type.

	Venous	Capillary	*P* value*
30% G6PD-deficient males and females, total study number N_D_	1,662	1,693	
Sensitivity (95% CI)	100.0 (93.6–100.0)	100.0 (93.8–100.0)	1.00
Specificity (95% CI)	98.6 (97.9–99.1)	97.8 (97.0–98.5)	0.05
70% G6PD-intermediate females, total study number N_I_	911	918	
Sensitivity (95% CI)	96.9 (83.8–99.9)	94.3 (80.8–99.3)	1.00
Specificity (95% CI)	96.5 (95.0–97.6)	92.3 (90.3–94.0)	0.01

G6PD, glucose-6-phosphate dehydrogenase; N_D_, total sample size for G6PD deficiency (both males and females); CI, confidence interval; N_I,_ total sample size for G6PD-intermediate performance (all females, not including G6PD-deficient females).

*McNemars *P* value comparing performance between venous and capillary specimens.

The sensitivity and specificity values in Table 4 describe the diagnostic performance for G6PD deficient and intermediate cases. False negatives were only observed for females with intermediate G6PD activity. From a clinical perspective, it is relevant to understand whether the false negatives arise from samples closer to the upper intermediate threshold (70% G6PD activity) or lower intermediate threshold (30% G6PD activity). Table 5 shows the performance of the 6.0 U/g Hb threshold on the STANDARD G6PD Test for identifying G6PD-deficient and G6PD-intermediate females with <70%, <65%, and <60% G6PD activity on the reference assay. Results are shown for capillary specimens. All specimens with less than 65% G6PD activity had a result on the STANDARD G6PD Test of less than or equal to 6.0 U/g Hb. Only two females with G6PD activity between 65% and 70% had a capillary STANDARD G6PD Test result of greater than 6.0 U/g Hb. For one of these two female participants, the test was run at an operating temperature greater than the maximum 40°C recommended by the manufacturer.

**Table 5:** Diagnostic performance of the 6.0 U/g Hb threshold on the STANDARD G6PD Test for all females on capillary specimens (n = 922). The sensitivity, specificity, and predictive power for all females with less than 70%, 65%, and 60% activity are shown.

	Reference G6PD activity		
	70%	65%	60%
**Sensitivity (95% CI)**	94.9 (82.7–99.4)	100.0 (88.8–100.0)	100.0 (88.1–100.0)
**Specificity (95% CI)**	92.3 (90.3–94.0)	91.7 (89.7–93.4)	91.5 (89.5–93.2)
**Positive predictive power (95% CI)**	35.2 (26.2–45.2)	29.5 (21.0–39.2)	27.6 (19.3–37.2)
**Negative predictive power (95% CI)**	99.8 (99.1–100.0)	100.0 (99.5–100.0)	100.0 (99.5–100.0)

CI, confidence interval; Hb, hemoglobin; G6PD, glucose-6-phosphate dehydrogenase.

The agreement between the result classification of the STANDARD G6PD Test and the spectrophotometric reference test was calculated and presented in 3x3 tables, by specimen type (Tables 6, 7, and S5). Overall, the STANDARD G6PD Test showed good agreement with the hemoglobin-normalized reference test result, at 96.7% for venous specimens (95% CI 95.7%–97.5%) and 94.1% for capillary specimens (95% CI 92.9%–95.2%).

**Table 6:** Percent agreement between the STANDARD G6PD Test and the spectrophotometric reference test using the manufacturer’s threshold values at 30% and 70% G6PD activity thresholds, on venous specimens.

		Spectrophotometric reference test			
		Deficient	Intermediate	Normal	Total
**STANDARD G6PD Test**	**Deficient**	56	14	9	79
	**Intermediate**	0	17	31	48
	**Normal**	0	1	1,534	1,535
	**Total**	56	32	1,574	1,662

G6PD, glucose-6-phosphate dehydrogenase.

Kappa: 0.73. Percent agreement between hemoglobin-normalized G6PD activity categorized results and the STANDARD Test was 96.7% (95% CI 95.7%–97.5%).

**Table 7:** Percent agreement between the STANDARD G6PD Test and the spectrophotometric reference test using the manufacturer’s threshold values at 30% and 70% G6PD activity thresholds, on capillary specimens.

		Spectrophotometric reference test			
		Deficient	Intermediate	Normal	Total
**STANDARD G6PD Test**	**Deficient**	58	17	19	94
	**Intermediate**	0	16	62	78
	**Normal**	0	2	1,519	1,521
	**Total**	58	35	1,600	1,693

G6PD, glucose-6-phosphate dehydrogenase.

Kappa: 0.60. Percent agreement between hemoglobin-normalized G6PD activity categorized results and the STANDARD Test was 94.1% (95% CI 92.9%–95.2%).

**Fig 3:**
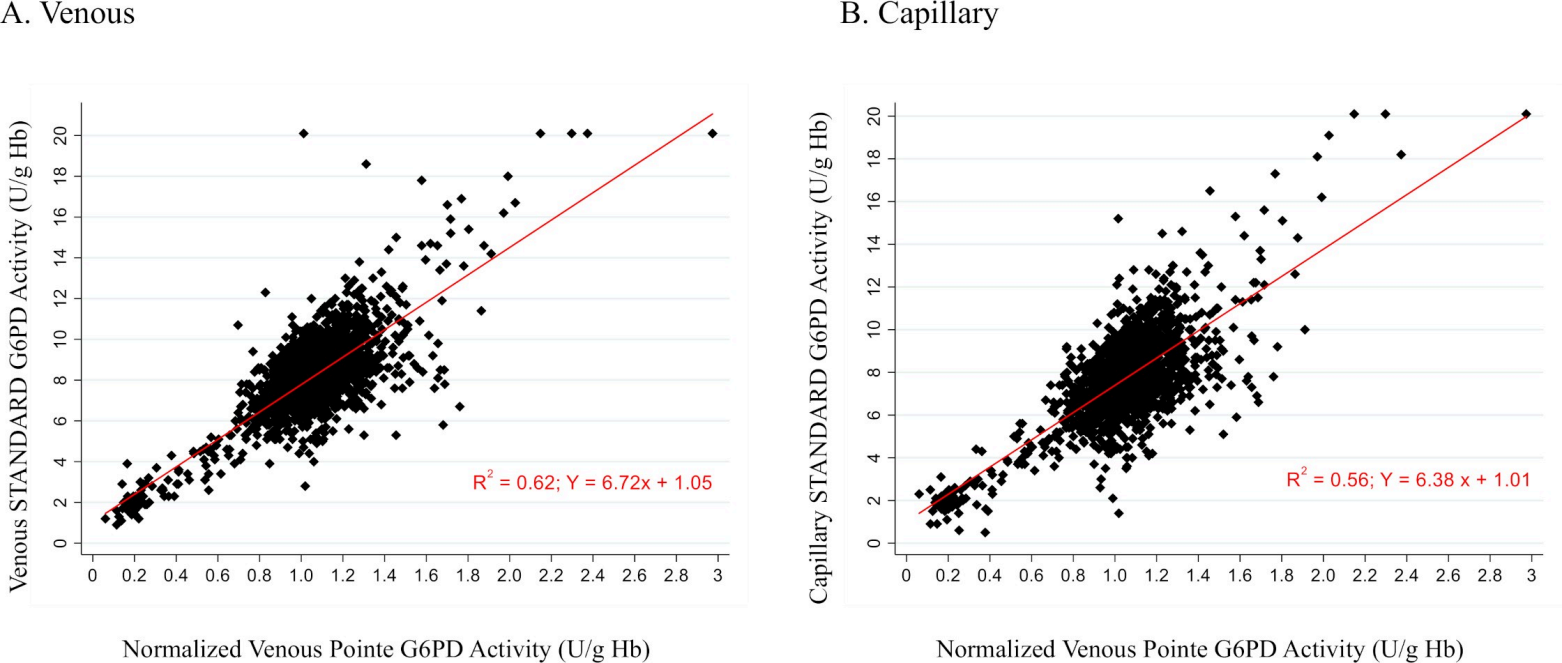
Regression analysis of STANDARD G6PD Test’s G6PD activity on A) venous specimens and B) capillary specimens, compared to normalized spectrophotometric reference test on venous specimens.

Linear regression of the STANDARD G6PD Test’s G6PD activity measurements as compared to the normalized reference assay results showed an R-squared correlation value of 0.62 for venous specimens and 0.56 for capillary specimens (Fig 3). The largest source of imprecision was observed in the high G6PD ranges, which lie within the normal range of G6PD activity. Bland-Altman plots are shown in supplemental materials for each site (S3 Fig).

### Clinical performance of the STANDARD G6PD Test for hemoglobin

**Fig 4:**
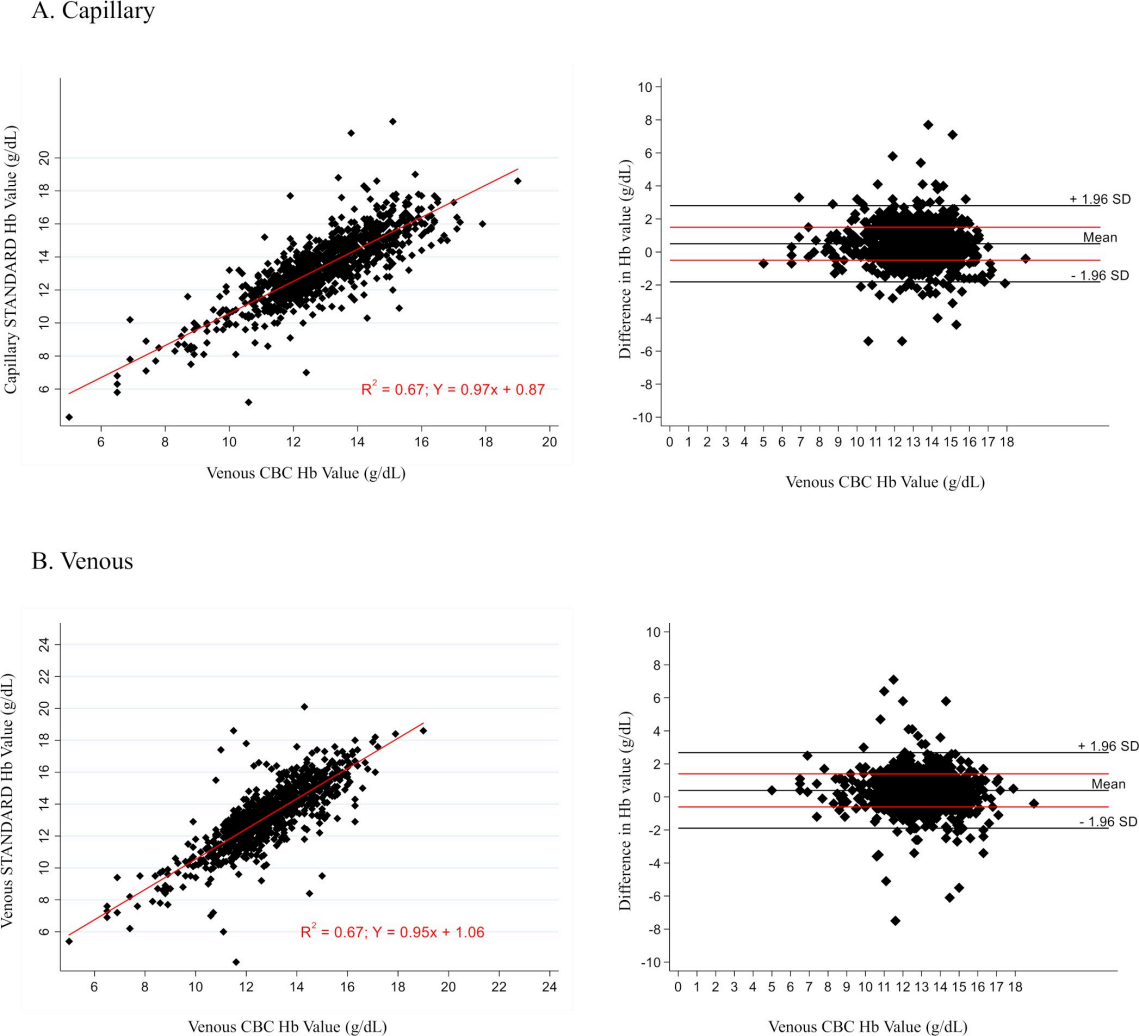
**Regression analysis of STANDARD G6PD Test total hemoglobin measurement on A) capillary specimens compared to complete blood count, B) venous specimens compared to complete blood count (Manaus only).** CBC, complete blood count; Hb, hemoglobin.

Linear regression of the STANDARD G6PD Test’s hemoglobin result as compared to the hemoglobin result from the complete blood count (CBC) on the venous K_2_EDTA blood sample in Manaus is shown in Fig 4, by specimen type. For both capillary and venous specimens, the R-squared correlation value was 0.67. The percent agreement was also assessed using the WHO definitions for anemia [35]. The percent agreement between the STANDARD G6PD Test hemoglobin measurement and the CBC on both capillary and venous specimens in Manaus was greater than 93% (S6 Table). Correlations of the HemoCue to the venous CBC hemoglobin results were higher than that of the STANDARD G6PD Test (S4 Fig), but the overall percent agreements were similar (S7 Table).

In Porto Velho, where hemoglobin determination by CBC was not possible and hemoglobin values are only available from the HemoCue, the STANDARD G6PD Test hemoglobin values were not evaluated for performance at that site. Correlations and agreement tables between the STANDARD G6PD Test and the HemoCue are provided in the supplemental materials (S5 Fig and S8 Table).

### G6PD deficiency and malaria

No significant difference in proportions of G6PD deficient and intermediates between microscopy positive and microscopy negative individuals in the study population was found with a Fisher’s exact test. Sub-analyses also explored the performance of the STANDARD G6PD Test by the malaria status of participants (S6 Fig; S9 Table). Correlation by linear regression of the STANDARD G6PD Test’s G6PD values compared to the spectrophotometric reference assay found that, for capillary specimens, the R-squared correlation value was 0.53 for malaria-negative participants as compared to 0.57 for malaria-positive participants. For venous specimens, the R-squared correlation was 0.60 and 0.54 for malaria-negative and malaria-positive participants, respectively. For G6PD-deficient males and females (<30% activity), the sensitivity and specificity of the STANDARD G6PD Test was slightly higher among malaria-positive participants as compared to malaria-negative participants on both capillary and venous specimens (S9 Table). For G6PD-intermediate females based on the 70% activity threshold, the sensitivity of the test was again slightly higher for malaria-positive participants; however, the test’s specificity was lower at 93.8% (95% CI 86.2%–98.0%) as compared to 96.7% (95% CI 95.3%–97.9%) for venous specimens, and 83.5% (95% CI 73.5%–90.9%) as compared to 93.2% (95% CI 91.2%–94.8%) for capillary specimens. All differences observed were within the 95% confidence intervals.

Further analysis also explored how results of the STANDARD G6PD Test might influence the administration of 8-aminoquiniline antimalarial medications among the study population. In total, 199 participants tested positive for *P*. *vivax* malaria by microscopy (N = 72 females; N = 127 males). Of these, one female and seven males tested G6PD deficient by the spectrophotometric reference assay, and three females tested G6PD intermediate using the 70% activity threshold (S10 Table). In comparison, results from the STANDARD G6PD Test on capillary samples taken at the point of care showed that two additional *P*. *vivax* patients, one male and one female, were classified as deficient, and 12 additional females were classified as intermediate. Of the 11 males with G6PD activity levels within intermediate ranges (30 to 70%) according to the reference test (Fig 1), none were malaria positive.

## Discussion

In malaria-endemic geographies such as Brazil, G6PD deficiency has important clinical implications for the radical cure of *P*. *vivax* malaria with 8-aminoquinoline drugs, which can lead to adverse health outcomes among patients with low G6PD activity [9]. Simple, affordable tests for G6PD deficiency that are appropriate for use where patients seek care for malaria will be critical in expanding access to safe treatments for radical cure [10]. Specifically, portable biosensor tests that provide quantitative result outputs for both G6PD and hemoglobin under a broad range of operating temperatures are particularly promising in their application to this use case due to their limited infrastructure requirements, coupled with their ability to identify heterozygous women with intermediate G6PD activity levels [36].

The STANDARD G6PD Test is a novel, point-of-care test for G6PD deficiency that provides a numeric measurement of G6PD activity normalized by hemoglobin. Previous studies have evaluated this test in the United States, Thailand, and Bangladesh, demonstrating promising analytical and clinical performance on both venous and capillary specimens with anti-coagulant [21,22].

This study focuses on the performance of the STANDARD G6PD Test in a malaria-endemic clinical setting in Brazil, with the majority of the recruitment conducted in outpatient clinics. The performance of the test was assessed on capillary samples at the point of care, as well as on venous K_2_EDTA specimens in a laboratory setting. Point-of-care testing was performed under a broad range of operating temperatures, ranging from 17.7°C to 43.7°C.

Results show that the STANDARD G6PD Test performed equivalently to the spectrophotometric reference test in the determination of G6PD status in venous and capillary whole blood across the dynamic range. At the 30% threshold for G6PD deficiency, the STANDARD G6PD Test demonstrated excellent performance on both capillary and venous specimens, with 100% sensitivity and >97% specificity for both sample types. In the context of malaria case management, the performance of the test among females with intermediate G6PD activity levels is of particular relevance due to the associated implications for both primaquine and Kozenis administration. Importantly, Kozenis is only indicated for patients with >70% G6PD activity—which must be established through the use of quantitative or semi-quantitative tests. At the 70% threshold, the STANDARD G6PD Test also demonstrated acceptable performance, with a sensitivity of 96.9% and 94.3% on venous and capillary specimens, respectively. Of note, two females with intermediate G6PD activity were misclassified as normal by the STANDARD G6PD Test on capillary specimens at this threshold. These cases are significant, as there is an associated risk that the patient and their provider may be unaware of the patient’s G6PD status and, as a result, the patient could be provided a contraindicated treatment, which might result in a clinically relevant adverse outcome. However, importantly, both of these participants with false negative results had G6PD activity levels of >65%, which is within the variability of the reference assay for G6PD activity [17]. Such misclassifications should be carefully monitored as part of any future studies evaluating test performance or implementation.

The ROC/AUC analysis for the performance of the STANDARD G6PD Test against the reference assay showed good discriminatory capacity of the test for G6PD-deficient individuals and females with intermediate G6PD activity both on capillary and venous specimens. Furthermore, the percent agreement with the reference assay for categorization of the G6PD-deficient, G6PD-intermediate, and G6PD-normal individuals using the manufacturer’s thresholds was good across both specimen types. Malaria status does not appear to impact the performance of the test. These findings support the feasibility of universal thresholds for use of the test at the point of care without the need to determine local G6PD reference ranges for the STANDARD G6PD Test.

Correlation between the STANDARD G6PD Test and the CBC in the measurement of hemoglobin was low, with R-squared values of 0.67 for both capillary and venous specimens. However, the STANDARD G6PD Test showed good categorical agreement with the CBC. Although this level of agreement was predominantly driven by the large proportion of non- and mild-anemia cases within the study population, the HemoCue showed similar patterns of misclassifications when compared to the CBC. For the STANDARD G6PD Test, confirmatory hemoglobin testing is indicated when monitoring disease progression or treatment decisions related to hemoglobin concentration.

The current primaquine dosages used for radical cure are considered safe in males and females not classified as G6PD deficient with widely used qualitative tests such as the FST, and more recently the CareStart G6PD test [19,37]. Higher dose regimens of primaquine—such as 1 mg/Kg/day for seven days—may be less safe for females with intermediate G6PD activity [36,38]. Given that Kozenis, the single-day regimen for radical cure, is indicated only for individuals with normal G6PD activity (greater than 70% activity), it should not be prescribed to intermediate females [14,39]. Among *P*. *vivax* positive patients in the study sample, the STANDARD G6PD Test overestimated the number with G6PD-deficient or intermediate activity levels on capillary specimens by n = 14 (12 normal females were classified as intermediates and two additional normal participants, one male and one female, were classified as deficient). This misclassification would likely have impacted the administration of 8-aminoquinolines to these individuals until confirmatory testing could be conducted. However, this risk should be considered in view of the overall benefits of increased safe availability of radical cure treatment options to people who otherwise may not have had access due to their unknown G6PD status.

A limitation of this study is the lower-than-expected number of enrolled participants with deficient and intermediate G6PD status. The 95% confidence intervals reported with the sensitivity and specificity values highlight this limitation. Further studies are required to better understand the performance of the test with intermediate females. Another limitation is the lack of available associated G6PD genotyping data which can further characterize specimens close to the critical thresholds.

## Conclusion

In summary, these data show that the STANDARD G6PD Test performed well in comparison to the spectrophotometric reference standard. The STANDARD G6PD Test is a promising tool to aid in the identification of G6PD deficiency in Brazil. The availability of this test represents an important opportunity to improve management of *P*. *vivax* malaria cases in resource-limited settings, where reliable alternatives to quantitative spectrophotometry are needed to expand access to radical cure. Future operational research should explore the feasibility and effectiveness of integrating point-of-care testing for G6PD deficiency into national malaria case management policies and practices.

## Supporting information

S1 Fig SD Biosensor STANDARD G6PD Test Capillary Quick Reference Guide. (PDF)

S2 Fig ROC curves at the 80% activity threshold on A) venous specimens and B) capillary specimens.
(DOCX)

S3 Fig Regression analyses and Bland-Altman plots of STANDARD G6PD Test’s G6PD activity on A) venous specimens compared to the spectrophotometric reference test in Manaus, B) venous specimens compared to the spectrophotometric reference test in Porto Velho, C) capillary specimens compared to the spectrophotometric reference test in Manaus, and D) capillary specimens compared to the spectrophotometric reference test in Porto Velho.
(DOCX)

S4 Fig Regression analysis and Bland-Altman plot of A) venous HemoCue total hemoglobin (T-Hb) measurement compared to venous complete blood count in Manaus, and B) capillary HemoCue T-Hb measurement compared to complete blood count in Manaus.
(DOCX)

S5 Fig Regression analysis and Bland-Altman plot of A) STANDARD G6PD Test total hemoglobin (T-Hb) measurement on venous specimens compared to venous HemoCue 201+, and B) STANDARD G6PD Test T-Hb measurement on capillary specimens compared to venous HemoCue 201+.
(DOCX)

S6 Fig Regression analyses and Bland-Altman plots of STANDARD G6PD Test activity compared to the spectrophotometric reference assay, by malaria status and specimen type for A) malaria negatives: capillary, B) malaria negatives: venous, C) malaria positives: capillary, and D) malaria positives: venous.
(DOCX)

S1 Table Descriptive statistics of the operating temperatures (degrees Celsius) under which the STANDARD G6PD Test was run, by specimen type and site. (DOCX)

S2 Table Areas under the curve (AUC) for receiver operating characteristics (ROC) analysis of the performance of the STANDARD G6PD Test for G6PD activity against the reference test for G6PD-deficient males and females as well as intermediate females, by specimen type. Two different definitions of intermediate females are shown.
(DOCX)

S3 Table Diagnostic performance of the STANDARD G6PD Test using the manufacturer’s thresholds compared with normalized spectrophotometric reference values at the 80% intermediate threshold, by specimen type. (DOCX)

S4 Table 2x2 tables for the clinical sensitivity and specificity of the STANDARD G6PD Test: A) for G6PD-deficient venous specimens, B) for female G6PD-intermediate venous specimens at 70%, C) for female G6PD-intermediate venous specimens at 80%, D) for G6PD-deficient capillary specimens, E) for female G6PD-intermediate capillary specimens at 70%, F) for female G6PD-intermediate capillary specimens at 80%.
(DOCX)

S5 Table Percent agreement using the manufacturer’s thresholds at 30% and 80% G6PD activity for A) venous specimens on the STANDARD Test compared to the spectrophotometric reference test and B) capillary specimens on the STANDARD Test compared to the spectrophotometric reference test.
(DOCX)

S6 Table Percent agreement for overall anemia status in Manaus between A) venous specimens on the STANDARD G6PD Test compared to complete blood count and B) capillary specimens on the STANDARD G6PD Test compared to complete blood count.
(DOCX)

S7 Table Percent agreement using overall anemia status in Manaus between A) venous specimens on the HemoCue compared to complete blood count and B) capillary specimens on the HemoCue compared to complete blood count.
(DOCX)

S8 Table Percent agreement using overall anemia status for A) venous specimens on the STANDARD G6PD Test compared to venous HemoCue and B) capillary specimens on the STANDARD G6PD Test compared to venous HemoCue.
(DOCX)

S9 Table Diagnostic performance analysis of the STANDARD G6PD Test with normalized spectrophotometric reference values and manufacturer thresholds, by malaria status and specimen type. (DOCX)

S10 Table G6PD status for participants with *P*. *vivax* malaria. (DOCX)
