# Supplementary material for: Evaluation of a point-of-care diagnostic to identify glucose-6-phosphate dehydrogenase deficiency in Brazil
Source: PLoS Negl Trop Dis. 2021 Aug 12;15(8):e0009649. doi: 10.1371/journal.pntd.0009649 (PMC8384181; doi:10.1371/journal.pntd.0009649)

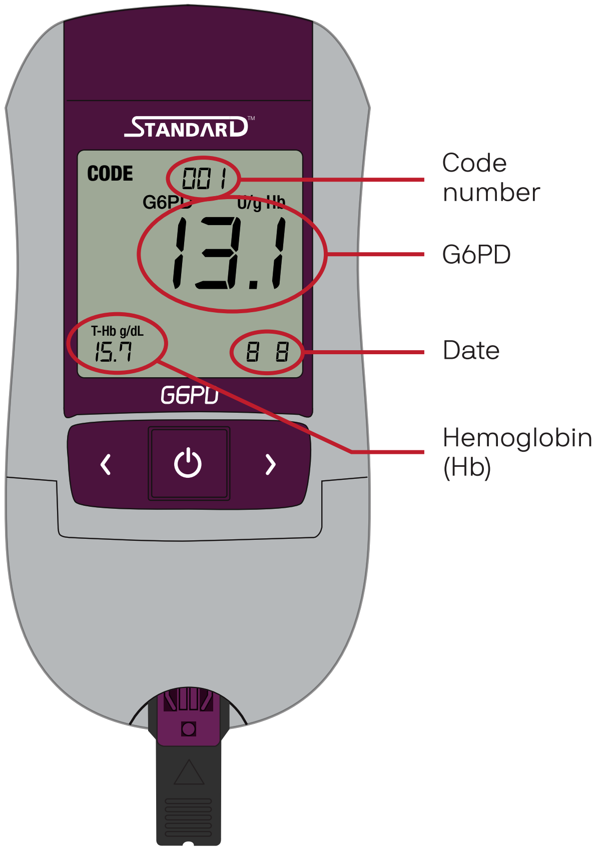

Intended use

The STANDARD G6PD Test measures quantitative G6PD enzyme activity and hemoglobin. The test can be used to identify people with low G6PD activity to inform treatment decisions at the point of care.

Components

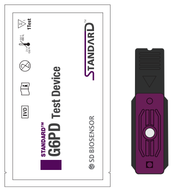

Test device

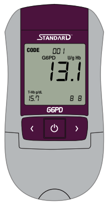

Analyzer

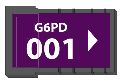

Code chip

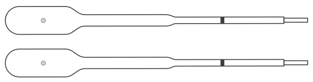

Sample collector (STANDARD™ Ezi Tube+)

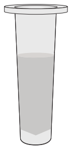

Extraction buffer

Test procedure

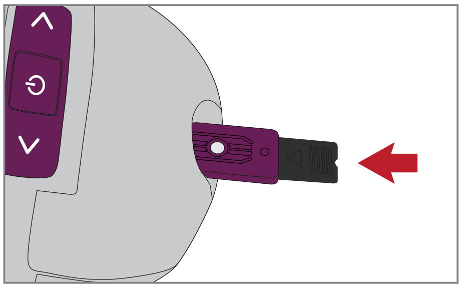

1. Insert test device into analyzer.

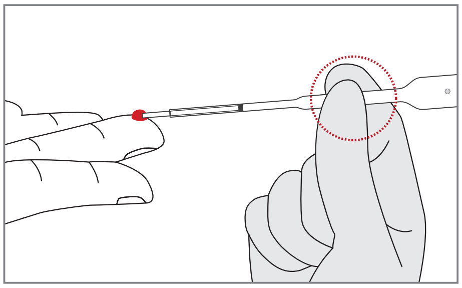

2. Collect blood.

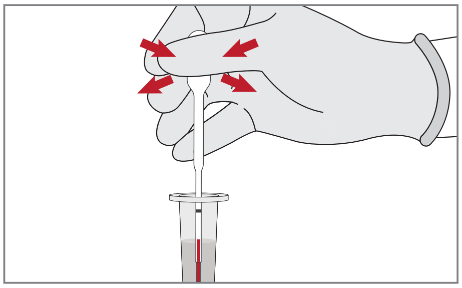

3. Mix blood and buffer 8–10 times.

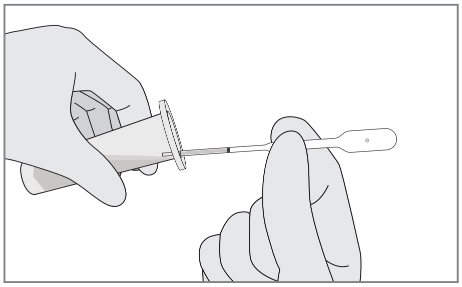

4. Collect mixed sample with NEW sample collector.

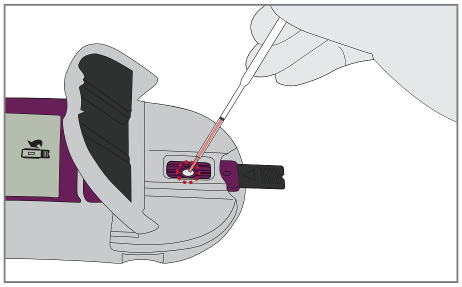

5. Apply the mixed sample to the hole in the test device.

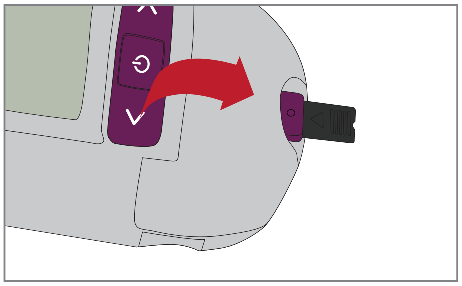

6. Close analyzer flap and wait for 2 minutes.

Results Interpretation

Classification	G6PD enzymatic activity (IU/g Hb)	
	Male	Female
Normal (high activity)	≥ 4.0	> 6.0
Intermediate (medium activity)	N/A	4.0–6.0
Deficient (low activity)	≤ 3.9	≤ 3.9

Remember to:

Check kit information.

Make sure the code number on the pouch and the code chip match. Do not use after the expiration date. Code chip must be inserted to perform test.

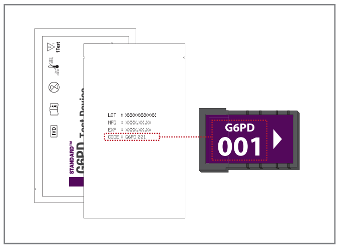

Collect sufficient amount of sample.

Ensure you collect sufficient volume of blood and buffer. Fill the sample collector to the black line.

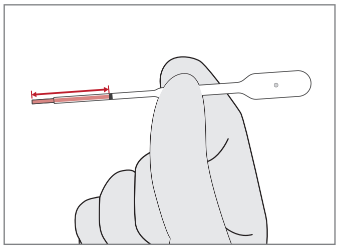

Use a new sample collector for each type of collection.

Use sample collector once for mixing blood with buffer and dispose it. For collecting the mixed sample, use the NEW sample collector.

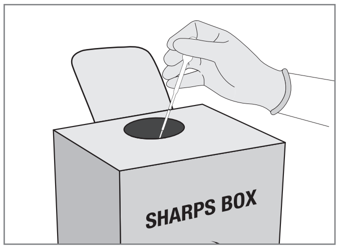

Close the analyzer flap immediately.

Close the analyzer flap immediately after applying mixed sample.

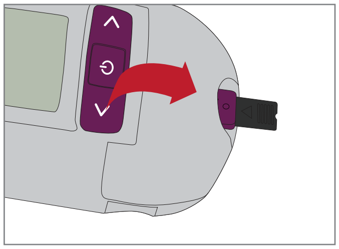

Read G6PD measurements and hemoglobin measurements separately.

For example, the top right 13.1 U/g Hb indicates units for G6PD measurement. The bottom left 15.7 T-Hb g/dL indicates hemoglobin measurement.

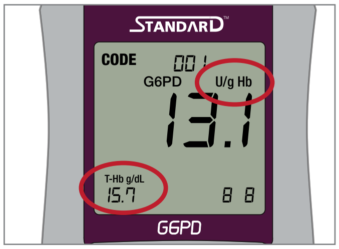

Supplement: S1 Fig — (PDF) [file pntd.0009649.s001.pdf]
