# Supplementary material for: Evaluation of a point-of-care diagnostic to identify glucose-6-phosphate dehydrogenase deficiency in Brazil
Source: PLoS Negl Trop Dis. 2021 Aug 12;15(8):e0009649. doi: 10.1371/journal.pntd.0009649 (PMC8384181; doi:10.1371/journal.pntd.0009649)
Supplement: S2 Fig — ROC curves at the 80% activity threshold on A) venous specimens and B) capillary specimens. (DOCX) [file pntd.0009649.s002.docx]

**Supplemental Fig S2**. ROC Curves at the 80% activity threshold on A) venous specimens, and B) capillary specimens.

A. Venous

B. Capillary

ROC, receiver operating characteristics.
