# Supplementary material for: Evaluation of a point-of-care diagnostic to identify glucose-6-phosphate dehydrogenase deficiency in Brazil
Source: PLoS Negl Trop Dis. 2021 Aug 12;15(8):e0009649. doi: 10.1371/journal.pntd.0009649 (PMC8384181; doi:10.1371/journal.pntd.0009649)
Supplement: S3 Fig — Regression analyses and Bland-Altman plots of STANDARD G6PD Test’s G6PD activity on A) venous specimens compared to the spectrophotometric reference test in Manaus, B) venous specimens compared to the spectrophotometric reference test in Porto Velho, C) capillary specimens compared to the spectrophotometric reference test in Manaus, and D) capillary specimens compared to the spectrophotometric reference test in Porto Velho. (DOCX) [file pntd.0009649.s003.docx]

**Supplemental Fig S3**. Regression analyses and Bland-Altman plots of STANDARD G6PD Test’s G6PD activity on A) venous specimens compared to the spectrophotometric reference test in Manaus, B) venous specimens compared to the spectrophotometric reference test in Porto Velho, C) capillary specimens compared to the spectrophotometric reference test in Manaus, and D) capillary specimens compared to the spectrophotometric reference test in Porto Velho.

A. Venous: Manaus

B. Venous: Porto Velho

C. Capillary: Manaus

D. Capillary: Porto Velho

G6PD, glucose-6-phosphate dehydrogenase; Hb, hemoglobin.
