# Supplementary material for: Evaluation of a point-of-care diagnostic to identify glucose-6-phosphate dehydrogenase deficiency in Brazil
Source: PLoS Negl Trop Dis. 2021 Aug 12;15(8):e0009649. doi: 10.1371/journal.pntd.0009649 (PMC8384181; doi:10.1371/journal.pntd.0009649)
Supplement: S4 Fig — Regression analysis and Bland-Altman plot of A) venous HemoCue total hemoglobin (T-Hb) measurement compared to venous complete blood count in Manaus, and B) capillary HemoCue T-Hb measurement compared to complete blood count in Manaus. (DOCX) [file pntd.0009649.s004.docx]

**Supplemental Fig S4**. Regression analysis and Bland-Altman plot of A) venous HemoCue total hemoglobin (T-Hb) measurement compared to venous complete blood count in Manaus and B) capillary HemoCue T-Hb measurement compared to complete blood count in Manaus.

A. Venous

B. Capillary

G6PD, glucose-6-phosphate dehydrogenase; Hb, hemoglobin.
