# Supplementary material for: Evaluation of a point-of-care diagnostic to identify glucose-6-phosphate dehydrogenase deficiency in Brazil
Source: PLoS Negl Trop Dis. 2021 Aug 12;15(8):e0009649. doi: 10.1371/journal.pntd.0009649 (PMC8384181; doi:10.1371/journal.pntd.0009649)
Supplement: S5 Fig — Regression analysis and Bland-Altman plot of A) STANDARD G6PD Test total hemoglobin (T-Hb) measurement on venous specimens compared to venous HemoCue 201+, and B) STANDARD G6PD Test T-Hb measurement on capillary specimens compared to venous HemoCue 201+. (DOCX) [file pntd.0009649.s005.docx]

**Supplemental Fig S5**. Regression analysis and Bland-Altman plot of A) STANDARD G6PD Test total hemoglobin (T-Hb) measurement on venous specimens compared to venous HemoCue 201+, and B) STANDARD G6PD Test T-Hb measurement on capillary specimens compared to venous HemoCue 201+.

A. Venous

B. Capillary

G6PD, glucose-6-phosphate dehydrogenase; Hb, hemoglobin.
