# Supplementary material for: Evaluation of a point-of-care diagnostic to identify glucose-6-phosphate dehydrogenase deficiency in Brazil
Source: PLoS Negl Trop Dis. 2021 Aug 12;15(8):e0009649. doi: 10.1371/journal.pntd.0009649 (PMC8384181; doi:10.1371/journal.pntd.0009649)
Supplement: S6 Fig — Regression analyses and Bland-Altman plots of STANDARD G6PD Test activity compared to the spectrophotometric reference assay, by malaria status and specimen type for A) malaria negatives: capillary, B) malaria negatives: venous, C) malaria positives: capillary, and D) malaria positives: venous. (DOCX) [file pntd.0009649.s006.docx]

**Supplemental Fig S6**. Regression analyses and Bland-Altman plots of STANDARD G6PD Test activity compared to the spectrophotometric reference assay, by malaria status and specimen type for A) malaria negatives: capillary, B) malaria negatives: venous, C) malaria positives: capillary, and D) malaria positives: venous.

A. Malaria negatives: capillary

B. Malaria negatives: venous

C. Malaria positives: capillary

D. Malaria positives: venous

G6PD: glucose-6-phosphate dehydrogenase

Hb: hemoglobin
