# Supplementary material for: Evaluation of a point-of-care diagnostic to identify glucose-6-phosphate dehydrogenase deficiency in Brazil
Source: PLoS Negl Trop Dis. 2021 Aug 12;15(8):e0009649. doi: 10.1371/journal.pntd.0009649 (PMC8384181; doi:10.1371/journal.pntd.0009649)
Supplement: S1 Table — (DOCX) [file pntd.0009649.s007.docx]

**Supplemental Table S1**. Descriptive statistics of the operating temperatures (degrees Celsius) under which the STANDARD G6PD Test was run, by specimen type and site.

|  | Manaus | Porto Velho | Combined |
| --- | --- | --- | --- |
| Venous |  |  |  |
| Mean temperature (SD) | 23.95 (2.02) | 22.42 (1.0) | 23.24 (1.79) |
| Median temperature | 23.70 | 22.34 | 22.80 |
| Range | 18.46–32.50 | 19.30–33.30 | 18.46–33.30 |
| Capillary |  |  |  |
| Mean temperature (SD) | 22.6 0 (3.34) | 24.89 (2.45) | 23.67 (3.18) |
| Median temperature | 21.98 | 24.50 | 23.40 |
| Range | 17.70–43.70 | 19.30–36.30 | 17.70–43.70 |

G6PD, glucose-6-phosphate dehydrogenase; SD, standard deviation.
