# Supplementary material for: Evaluation of a point-of-care diagnostic to identify glucose-6-phosphate dehydrogenase deficiency in Brazil
Source: PLoS Negl Trop Dis. 2021 Aug 12;15(8):e0009649. doi: 10.1371/journal.pntd.0009649 (PMC8384181; doi:10.1371/journal.pntd.0009649)
Supplement: S2 Table — Two different definitions of intermediate females are shown. (DOCX) [file pntd.0009649.s008.docx]

**Supplemental Table S2**. Area under the curve (AUC) for receiver operating characteristics (ROC) analysis of the performance of the STANDARD G6PD Test for G6PD activity against the reference test for G6PD-deficient males and females as well as intermediate females, by specimen type. Two different definitions of intermediate females are shown.

| **STANDARD G6PD Test activity (U/g Hb)** | **N** | **AUC (95%CI)** |
| --- | --- | --- |
| **Venous** |  |  |
| ≤30% activity males and females (deficient) | 56 | 1.00 (0.99 – 1.00) |
| >30% to ≤70% activity females only (intermediate) | 61 | 1.00 (0.99 – 1.00) |
| >30% to ≤80% activity females only (intermediate) | 32 | 0.95 (0.92 – 0.98)* |
| **Capillary** |  |  |
| ≤30% activity males and females(deficient) | 58 | 1.00 (0.99 – 1.00) |
| >30% to ≤70% activity females only (intermediate) | 64 | 0.98 (0.96 – 1.00) |
| >30% to ≤80% activity females only(intermediate) | 35 | 0.90 (0.86 – 0.95)* |

AUC, area under the curve; ROC, receiver operating characteristic; G6PD, glucose-6-phosphate dehydrogenase; Hb, hemoglobin.

**P* value for comparison of AUC curves between venous and capillary at 80% = 0.01
