# Supplementary material for: Evaluation of a point-of-care diagnostic to identify glucose-6-phosphate dehydrogenase deficiency in Brazil
Source: PLoS Negl Trop Dis. 2021 Aug 12;15(8):e0009649. doi: 10.1371/journal.pntd.0009649 (PMC8384181; doi:10.1371/journal.pntd.0009649)
Supplement: S3 Table — (DOCX) [file pntd.0009649.s009.docx]

**Supplemental Table S3**. Diagnostic performance of the STANDARD G6PD Test using the manufacturer’s thresholds compared with normalized spectrophotometric reference values at the 80% intermediate threshold, by specimen type.

|  | **Venous** | **Capillary** |
| --- | --- | --- |
| 80% intermediate females, total study number N_I_ | 911 | 918 |
| Sensitivity (95% CI) | 67.2 (54.0–78.7) | 67.2 (54.3–78.4) |
| Specificity (95% CI) | 97.5 (96.2–98.5) | 93.2 (91.3–94.8) |

N_I,_ total sample size for G6PD intermediate performance (all females, not including G6PD-deficient females); CI, confidence interval.
