# Supplementary material for: Evaluation of a point-of-care diagnostic to identify glucose-6-phosphate dehydrogenase deficiency in Brazil
Source: PLoS Negl Trop Dis. 2021 Aug 12;15(8):e0009649. doi: 10.1371/journal.pntd.0009649 (PMC8384181; doi:10.1371/journal.pntd.0009649)
Supplement: S4 Table — 2x2 tables for the clinical sensitivity and specificity of the STANDARD G6PD Test: A) for G6PD-deficient venous specimens, B) for female G6PD-intermediate venous specimens at 70%, C) for female G6PD-intermediate venous specimens at 80%, D) for G6PD-deficient capillary specimens, E) for female G6PD-intermediate capillary specimens at 70%, F) for female G6PD-intermediate capillary specimens at 80%. (DOCX) [file pntd.0009649.s010.docx]

**Supplemental Table S4**. 2x2 tables for the clinical sensitivity and specificity of the STANDARD G6PD Test: A) for G6PD-deficient venous specimens, B) for female G6PD-intermediate venous specimens at 70%, C) for female G6PD-intermediate venous specimens at 80%, D) for G6PD-deficient capillary specimens, E) for female G6PD-intermediate capillary specimens at 70%, and F) for female G6PD-intermediate capillary specimens at 80%.

A. G6PD-deficient venous specimens

|  | **True positive** | **True negative** | **Total** |
| --- | --- | --- | --- |
| **STANDARD G6PD deficient** | 56 | 23 | 79 |
| **STANDARD G6PD normal or intermediate** | 0 | 1,583 | 1,583 |
| **Total** | 56 | 1,606 | 1,662 |

B. Female G6PD-intermediate venous specimens at 70%

|  | **True positive** | **True negative** | **Total** |
| --- | --- | --- | --- |
| **STANDARD G6PD deficient** | 31 | 31 | 62 |
| **STANDARD G6PD normal or intermediate** | 1 | 848 | 849 |
| **Total** | 32 | 879 | 911 |

C. Female G6PD-intermediate venous specimens at 80%

|  | **True positive** | **True negative** | **Total** |
| --- | --- | --- | --- |
| **STANDARD G6PD deficient** | 41 | 21 | 62 |
| **STANDARD G6PD normal or intermediate** | 20 | 829 | 849 |
| **Total** | 61 | 850 | 911 |

D. G6PD-deficient capillary specimens

|  | **True positive** | **True negative** | **Total** |
| --- | --- | --- | --- |
| **STANDARD G6PD deficient** | 58 | 36 | 94 |
| **STANDARD G6PD normal or intermediate** | 0 | 1,599 | 1,599 |
| **Total** | 58 | 1,635 | 1,693 |

E. Female G6PD-intermediate capillary specimens at 70%

|  | **True positive** | **True negative** | **Total** |
| --- | --- | --- | --- |
| **STANDARD G6PD deficient** | 33 | 68 | 101 |
| **STANDARD G6PD normal or intermediate** | 2 | 815 | 817 |
| **Total** | 35 | 883 | 918 |

F. Female G6PD-intermediate capillary specimens at 80%

|  | **True positive** | **True negative** | **Total** |
| --- | --- | --- | --- |
| **STANDARD G6PD deficient** | 43 | 58 | 101 |
| **STANDARD G6PD normal or intermediate** | 21 | 796 | 817 |
| **Total** | 64 | 817 | 918 |

G6PD, glucose-6-phosphate dehydrogenase.
