# Supplementary material for: Evaluation of a point-of-care diagnostic to identify glucose-6-phosphate dehydrogenase deficiency in Brazil
Source: PLoS Negl Trop Dis. 2021 Aug 12;15(8):e0009649. doi: 10.1371/journal.pntd.0009649 (PMC8384181; doi:10.1371/journal.pntd.0009649)
Supplement: S5 Table — Percent agreement using the manufacturer’s thresholds at 30% and 80% G6PD activity for A) venous specimens on the STANDARD Test compared to the spectrophotometric reference test and B) capillary specimens on the STANDARD Test compared to the spectrophotometric reference test. (DOCX) [file pntd.0009649.s011.docx]

**Supplemental Table S5**. Percent agreement using the manufacturer’s thresholds at 30% and 80% G6PD activity for A) venous specimens on the STANDARD Test compared to the spectrophotometric reference test and B) capillary specimens on the STANDARD Test compared to the spectrophotometric reference test.

A. Venous

|  | | **Spectrophotometric reference test** | | |  |
| --- | --- | --- | --- | --- | --- |
|  |  | **Deficient** | **Intermediate** | **Normal** | **Total** |
| **STANDARD G6PD Test** | **Deficient** | 56 | 14 | 9 | 79 |
|  | **Intermediate** | 0 | 27 | 21 | 48 |
|  | **Normal** | 0 | 20 | 1,515 | 1,535 |
|  | **Total** | 56 | 61 | 1,545 | 1,662 |

Percent agreement between hemoglobin-normalized G6PD activity categorized results and the STANDARD test was 96.2% (95% CI: 95.1%–97.0%).

B. Capillary

|  | | **Spectrophotometric reference test** | | |  |
| --- | --- | --- | --- | --- | --- |
|  |  | **Deficient** | **Intermediate** | **Normal** | **Total** |
| **STANDARD G6PD Test** | **Deficient** | 58 | 18 | 18 | 94 |
|  | **Intermediate** | 0 | 25 | 53 | 78 |
|  | **Normal** | 0 | 21 | 1,500 | 1,521 |
|  | **Total** | 58 | 64 | 1,571 | 1,693 |

Percent agreement between hemoglobin-normalized G6PD activity categorized results and the STANDARD Test was 93.5% (95% CI: 92.2%–94.6%).
