# Supplementary material for: Evaluation of a point-of-care diagnostic to identify glucose-6-phosphate dehydrogenase deficiency in Brazil
Source: PLoS Negl Trop Dis. 2021 Aug 12;15(8):e0009649. doi: 10.1371/journal.pntd.0009649 (PMC8384181; doi:10.1371/journal.pntd.0009649)
Supplement: S6 Table — Percent agreement for overall anemia status in Manaus between A) venous specimens on the STANDARD G6PD Test compared to complete blood count and B) capillary specimens on the STANDARD G6PD Test compared to complete blood count. (DOCX) [file pntd.0009649.s012.docx]

**Supplemental Table S6**. Percent agreement for overall anemia status in Manaus between A) venous specimens on the STANDARD G6PD Test compared to complete blood count and B) capillary specimens on the STANDARD G6PD Test compared to complete blood count.

A. Venous

|  | | **Complete blood count (CBC)** | | | |
| --- | --- | --- | --- | --- | --- |
|  |  | **Non/mild anemia** | **Moderate anemia** | **Severe**  **anemia** | **Total** |
| **STANDARD G6PD Test** | **Non/mild anemia** | 731 | 22 | 0 | 753 |
|  | **Moderate anemia** | 28 | 52 | 2 | 82 |
|  | **Severe anemia** | 2 | 5 | 8 | 15 |
|  | **Total** | 761 | 79 | 10 | 850 |

Percent agreement between CBC and the STANDARD Test was 93.1% (95% CI: 91.1%–94.7%).

B. Capillary

|  | | **Complete blood count (CBC)** | | | |
| --- | --- | --- | --- | --- | --- |
|  |  | **Non/mild anemia** | **Moderate anemia** | **Severe**  **anemia** | **Total** |
| **STANDARD G6PD Test** | **Non/mild anemia** | 784 | 30 | 0 | 814 |
|  | **Moderate anemia** | 25 | 48 | 3 | 76 |
|  | **Severe anemia** | 1 | 2 | 7 | 10 |
|  | **Total** | 810 | 80 | 10 | 900 |

Percent agreement between CBC and the STANDARD Test was 93.2% (95% CI: 91.4%–94.8%).
