# Supplementary material for: Evaluation of a point-of-care diagnostic to identify glucose-6-phosphate dehydrogenase deficiency in Brazil
Source: PLoS Negl Trop Dis. 2021 Aug 12;15(8):e0009649. doi: 10.1371/journal.pntd.0009649 (PMC8384181; doi:10.1371/journal.pntd.0009649)
Supplement: S7 Table — Percent agreement using overall anemia status in Manaus between A) venous specimens on the HemoCue compared to complete blood count and B) capillary specimens on the HemoCue compared to complete blood count. (DOCX) [file pntd.0009649.s013.docx]

**Supplemental Table S7**. Percent agreement using overall anemia status in Manaus between A) venous specimens on the HemoCue compared to complete blood count and B) capillary specimens on the HemoCue compared to complete blood count.

A. Venous

|  | | **Complete blood count (CBC)** | | | |
| --- | --- | --- | --- | --- | --- |
|  |  | **Non/mild anemia** | **Moderate anemia** | **Severe**  **anemia** | **Total** |
| **HemoCue** | **Non/mild anemia** | 747 | 33 | 1 | 781 |
|  | **Moderate anemia** | 13 | 44 | 1 | 58 |
|  | **Severe anemia** | 1 | 2 | 8 | 11 |
|  | **Total** | 761 | 79 | 10 | 850 |

Percent agreement between CBC and the HemoCue test was 94.0% (95% CI: 92.2%–95.5%).

B. Capillary

|  | | **Complete blood count (CBC)** | | | |
| --- | --- | --- | --- | --- | --- |
|  |  | **Non/mild anemia** | **Moderate anemia** | **Severe**  **anemia** | **Total** |
| **HemoCue** | **Non/mild anemia** | 784 | 39 | 2 | 825 |
|  | **Moderate anemia** | 7 | 41 | 4 | 52 |
|  | **Severe anemia** | 0 | 0 | 4 | 4 |
|  | **Total** | 791 | 80 | 10 | 881 |

Percent agreement between CBC and the HemoCue test was 94.1% (95% CI: 92.3%–95.6%).
