# Supplementary material for: Evaluation of a point-of-care diagnostic to identify glucose-6-phosphate dehydrogenase deficiency in Brazil
Source: PLoS Negl Trop Dis. 2021 Aug 12;15(8):e0009649. doi: 10.1371/journal.pntd.0009649 (PMC8384181; doi:10.1371/journal.pntd.0009649)
Supplement: S8 Table — Percent agreement using overall anemia status for A) venous specimens on the STANDARD G6PD Test compared to venous HemoCue and B) capillary specimens on the STANDARD G6PD Test compared to venous HemoCue. (DOCX) [file pntd.0009649.s014.docx]

**Supplemental Table S8**. Percent agreement using overall anemia status for A) venous specimens on the STANDARD G6PD Test compared to venous HemoCue and B) capillary specimens on the STANDARD G6PD Test compared to venous HemoCue.

A. Venous

|  | | **HemoCue** | | | |
| --- | --- | --- | --- | --- | --- |
|  |  | **Non/mild anemia** | **Moderate anemia** | **Severe**  **anemia** | **Total** |
| **STANDARD G6PD Test** | **Non/mild anemia** | 1,480 | 46 | 1 | 1,527 |
|  | **Moderate anemia** | 57 | 60 | 2 | 119 |
|  | **Severe anemia** | 2 | 3 | 11 | 16 |
|  | **Total** | 1,539 | 109 | 14 | 1,662 |

Percent agreement between HemoCue and the STANDARD Test was 93.3% (95% CI: 92.0%–94.5%).

B. Capillary

|  | | **HemoCue** | | | |
| --- | --- | --- | --- | --- | --- |
|  |  | **Non/mild anemia** | **Moderate anemia** | **Severe**  **anemia** | **Total** |
| **STANDARD G6PD Test** | **Non/mild anemia** | 1,475 | 35 | 2 | 1,512 |
|  | **Moderate anemia** | 93 | 71 | 4 | 168 |
|  | **Severe anemia** | 3 | 2 | 8 | 13 |
|  | **Total** | 1,571 | 108 | 14 | 1,693 |

Percent agreement between HemoCue and the STANDARD Test was 91.8% (95% CI: 90.4%–93.1%).

G6PD, glucose-6-phosphate dehydrogenase.
