# Supplementary material for: Evaluation of a point-of-care diagnostic to identify glucose-6-phosphate dehydrogenase deficiency in Brazil
Source: PLoS Negl Trop Dis. 2021 Aug 12;15(8):e0009649. doi: 10.1371/journal.pntd.0009649 (PMC8384181; doi:10.1371/journal.pntd.0009649)
Supplement: S9 Table — (DOCX) [file pntd.0009649.s015.docx]

**Supplemental Table S9**. Diagnostic performance analysis of the STANDARD G6PD Test with normalized spectrophotometric reference values and manufacturer thresholds, by malaria status and specimen type.

|  | **Malaria negatives** | **Malaria positives** |
| --- | --- | --- |
| **Venous** |  |  |
| **30% G6PD-deficient males and females, total study number N_D_** | 1,415 | 246 |
| Sensitivity (95% CI) | 100.0  (92.1–100.0) | 100.0  (71.5–100.0) |
| Specificity (95% CI) | 98.5  (97.7–99.0) | 99.1  (97.0–99.9) |
| **70% G6PD-intermediate females, total study number N_I_** | 826 | 85 |
| Sensitivity (95% CI) | 96.4  (81.7–99.9) | 100.0  (39.8–100.0) |
| Specificity (95% CI) | 96.7  (95.3–97.9) | 93.8  (86.2–98.0) |
| **Capillary** |  |  |
| **30% G6PD-deficient males and females, total study number N_D_** | 1,451 | 242 |
| Sensitivity (95% CI) | 100.0  (92.5–100.0) | 100.0  (71.5–100.0) |
| Specificity (95% CI) | 97.7  (96.8–98.4) | 98.3  (95.6–99.5) |
| **70% G6PD-intermediate females, total study number N_I_** | 835 | 83 |
| Sensitivity (95% CI) | 93.5  (78.6–99.2) | 100.0  (39.8–100.0) |
| Specificity (95% CI) | 93.2  (91.2–94.8) | 83.5  (73.5–90.9) |

G6PD, glucose-6-phosphate dehydrogenase; N_D_, total sample size for G6PD deficiency (both males and females); CI, confidence interval; N_I,_ total sample size for G6PD intermediate performance (all females, not including G6PD deficient females).
