# Supplementary material for: Evaluation of a point-of-care diagnostic to identify glucose-6-phosphate dehydrogenase deficiency in Brazil
Source: PLoS Negl Trop Dis. 2021 Aug 12;15(8):e0009649. doi: 10.1371/journal.pntd.0009649 (PMC8384181; doi:10.1371/journal.pntd.0009649)
Supplement: S10 Table — (DOCX) [file pntd.0009649.s016.docx]

**Supplemental Table S10**. G6PD status for participants with *P. vivax* malaria.

|  | **G6PD status, females (N=72)**  **(spectrophotometric reference assay, venous)*** | | | **G6PD status, males (N=127)**  **(spectrophotometric reference assay, venous)*** | | |
| --- | --- | --- | --- | --- | --- | --- |
|  | Deficient | Intermediate | Normal | Deficient | Intermediate | Normal |
| **G6PD status**  **(STANDARD G6PD Test, venous)** |  |  |  |  |  |  |
| Deficient | 1 | 0 | 0 | 7 | 0 | 0 |
| Intermediate | 0 | 3 | 4 | 0 | 0 | 0 |
| Normal | 0 | 0 | 64 | 0 | 0 | 120 |
| **G6PD status**  **(STANDARD G6PD Test, capillary)** |  |  |  |  |  |  |
| Deficient | 1 | 0 | 1 | 7 | 0 | 1 |
| Intermediate | 0 | 3 | 12 | 0 | 0 | 0 |
| Normal | 0 | 0 | 55 | 0 | 0 | 119 |

G6PD, glucose-6-phosphate dehydrogenase.

*based on 30% and 70% activity thresholds on the spectrophotometric reference assay.
